# A Novel Method for *In-Situ* Monitoring of Local Voltage, Temperature and Humidity Distributions in Fuel Cells Using Flexible Multi-Functional Micro Sensors

**DOI:** 10.3390/s110201418

**Published:** 2011-01-26

**Authors:** Chi-Yuan Lee, Wei-Yuan Fan, Chih-Ping Chang

**Affiliations:** Department of Mechanical Engineering, Yuan Ze Fuel Cell Center, Yuan Ze University, Taoyuan, Taiwan; E-Mails: s975040@mail.yzu.edu.tw (W.-Y.F.); s995042@mail.yzu.edu.tw (C.-P.C.)

**Keywords:** MEMS, PEMFC, Flexible multi-functional micro sensors

## Abstract

In this investigation, micro voltage, temperature and humidity sensors were fabricated and integrated for the first time on a stainless steel foil using micro-electro-mechanical systems (MEMS). These flexible multi-functional micro sensors have the advantages of high temperature resistance, flexibility, smallness, high sensitivity and precision of location. They were embedded in a proton exchange membrane fuel cell (PEMFC) and used to simultaneously measure variations in the inner voltage, temperature and humidity. The accuracy and reproducibility of the calibrated results obtained using the proposed micro sensors is excellent. The experimental results indicate that, at high current density and 100%RH or 75%RH, the relative humidity midstream and downstream saturates due to severe flooding. The performance of the PEM fuel cell can be stabilized using home-made flexible multi-functional micro sensors by the *in-situ* monitoring of local voltage, temperature and humidity distributions within it.

## 1. Introduction

Global warming and energy crises have accelerated the search for sustainable alternative sources of power. Despite their environmental friendliness, high energy conversion efficiency, low noise, and wide applicability, fuel cells are still limited by their prohibitively high cost, membrane degradation, and the difficulty of monitoring them and diagnosing problems.

The distribution of voltage, temperature and humidity in a proton exchange membrane fuel cell (PEMFC) are critical factors that influence cell performance. The conductivity and water content of the proton exchange membrane directly affects performance [1,2], because excess water in the flow channel can cause flooding and prevent gas diffusion: excessively high or low temperatures can then cause dehydration of the proton exchange membrane, and both worsen fuel cell performance. Liu demonstrated that the accumulation of liquid water columns in the cathode flow channels reduces the effective electrochemical reaction area, limiting mass transfer and worsening cell performance [3]. Wang noted that liquid water management significantly affects PEMFC performance, especially at high current density [4]. Therefore, suitable water and thermal management should be used to ensure that the proton exchange membrane is sufficiently hydrated to maintain high proton conductivity.

Li reviewed more than 100 references related to water management in proton exchange membrane fuel cells (PEMFCs), with a particular focus on water flooding, its diagnosis and mitigation [5]. Trabold applied neutron imaging to research the distribution of water flooding, detecting *in situ* variation in the amount of water that is produced in an operating fuel cell [6]. Tests that were performed by Zhang revealed that performance gradually worsened as relative humidity declined from 100% to 25% [7].

Most investigations of voltage and humidity in PEMFCs involve the insertion of small sensors into the cells. For example, David examined the temperature distribution in fuel cells using Fiber Bragg grating technology. The result revealed a difference between the temperatures of the inlet and the outlet of 1 °C [8]. Inman measured *in-situ* the reaction temperature in an operating fuel cell by placing five fiber temperature sensors in it [9]. Hinds employed commercial temperature and humidity sensors, with a large active area, in a single cell PEMFC [2]. Nishikawa cut the flow channel plate to install a commercial humidity sensor. This method yielded information about the interior, but the cost and assembly were problematic [10].

Wang utilized an infrared temperature device to measure external temperature distribution under various operating conditions [11]. Karimi observed the distribution of water within fuel cell stacks. His simulation results revealed that increasing the humidity promoted water flooding downstream [12]. Shimpalee simulated variations in temperature, humidity, and current in a PEMFC. His results demonstrated that water flooding downstream affected the fuel cell reaction, indirectly reducing the temperature and current [13].

In the aforementioned references, bipolar plates were cut and processed, and then sensors were inserted into fuel cells to measure internal physical values. This process can not only cause fuel leakage but also increase contact resistance. Along with invasive measurement, simulation can also identify water flooding. However, neither of these methods can be used to obtain accurate information on the interiors of fuel cells.

Lee embedded micro flexible micro sensors in a membrane electrode assembly (MEA) to measure the temperature and humidity in a micro fuel cell [14]. Lee also successfully measured the local voltage and temperature in a PEMFC using micro voltage and temperature sensors [15]. The present investigation proposes a novel method for fabricating flexible multi-functional micro sensors of voltage, temperature and humidity on a stainless steel foil substrate using micro-electro-mechanical systems (MEMS). Home-made flexible multi-functional micro sensors have numerous advantages, including high temperature resistance, smallness, high sensitivity, and precision of measurement position. Flexible multi-functional micro sensors were embedded in a cathode flow channel of a PEMFC to measure and analyze variations in internal local voltage, temperature and humidity, and then to identify water flooding.

## 2. Theory and Design of Micro Sensors

### 2.1. Micro Voltage Sensor

**Figure 1.**
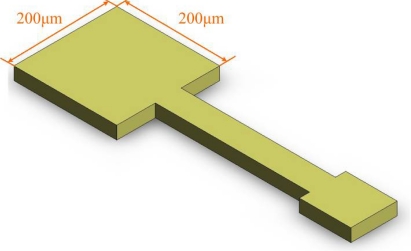
Design of the micro voltage sensor.

The voltage sensor that was used herein is a miniaturized voltage probe. This film-type probe is embedded inside a fuel cell to take measurements in particular locations. The sensing area of the voltage sensor, in contact with the bipolar plate of the fuel cell, is 200 μm × 200 μm. The rest of the conducting wire is insulated. Figure 1 displays the micro voltage sensor.

### 2.2. Micro Temperature Sensor

**Figure 2.**
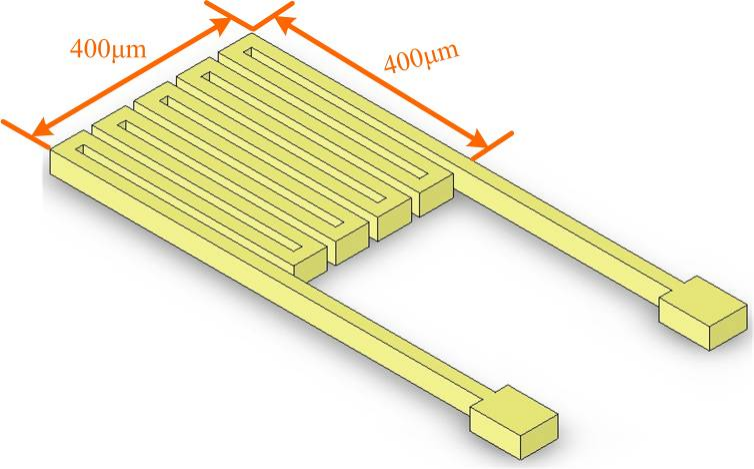
Design of the micro temperature sensor.

The temperature sensor utilized herein is a resistance temperature detector (RTD), which has the advantages of a large range of sensing temperatures and high linearity. The electrodes had serpentine structures, with a sensing area of 400 μm × 400 μm, as shown in Figure 2.

An increase in the environmental temperature increases the resistance of the RTD because a metal conductor has a positive temperature coefficient (PTC). When the temperature of the RTD varies in the linear region, the relationship between the measured resistance and the change in temperature can be expressed as:

(1) $$R_{t}=R_{r}\;(1+\alpha_{T}\Delta T)$$

where R_t_ is the resistance at t °C; R_r_ is the resistance at i °C, and *α_T_* is the sensitivity (1/°C) [16].

### 2.3. Micro Humidity Sensor

Wang measured the humidity of silicon nitride ceramics using a capacitive humidity sensor. The time to respond from high to low humidity was short [17]. Chang fabricated zinc oxide nanowires on a silicon chip, in the form of a resistive humidity sensor, to sense humidity [18]. However, in both approaches, the fabrication was highly complex and neither scheme is suitable for fuel cells.

Laconte fabricated a capacitive humidity sensor from a complementary metal-oxide semiconductor (CMOS). A coat of polyimide was deposited on interdigitated electrodes. This approach greatly improved the sensitivity and response time of the sensors [19]. Fürjes fabricated a heater around the humidity sensor, and his results revealed that the use of a heater shortened the measurement time from the original 15 minutes to 20 s [20].

A capacitive humidity sensor of interdigitated electrodes is adopted herein, because such a sensor is less affected by temperature than is a resistive one. The humidity-sensing film is polyimide, because its chemical properties are stable and it has a high temperature tolerance. As the amount of steam that is absorbed by the polyimide increases, the dielectric constant increases and the increase in capacitance is given by Equation (2),

(2) $$C={ɛ}_{0}{ɛ}_{r}\frac{A}{d}$$

where *C* represents capacitance (F); *ɛ*_0_ is the dielectric constant in a vacuum; *ɛ_r_* is the dielectric constant of the environment; *A* is the area of the electrode (m^2^), and *d* is the distance between the two electrodes (m).

**Figure 3.**
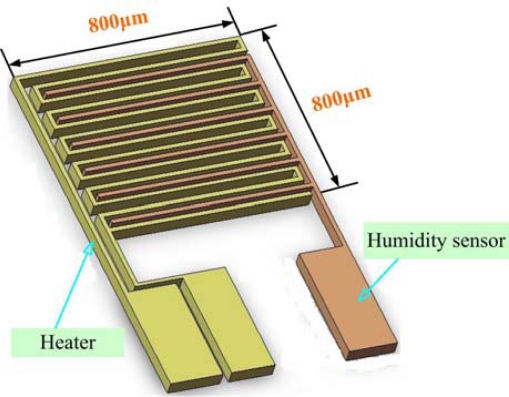
Design of the micro humidity sensor.

A humidity-sensing film absorbs moisture easily but does not allow that moisture to be easily removed. However, the use of a micro heater can eliminate this problem, which causes the response time of micro humidity sensors to be long. Figure 3 shows the overall design of a micro humidity sensor.

## 3. Fabrication of Flexible Multi-Functional Micro Sensors

In this study, flexible multi-functional micro sensors that sense voltage, temperature and humidity were integrated on stainless steel foil (SS304, 40 μm thick) using micro-electro-mechanical systems (MEMS). Stainless steel foils have numerous favorable properties, including high corrosion resistance, high compression resistance, high temperature resistance, and high flexibility.

**Figure 4.**
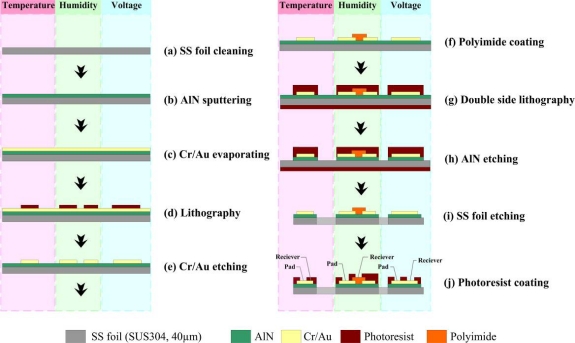
Fabrication of flexible multi-functional micro sensors.

Figure 4 presents the flowchart for the fabrication of flexible multi-functional micro sensors. The steps are as follows. (a) Use acetone and methanol to clean any grease from the surface of the stainless steel foil, and then use a mixed solvent of sulfuric acid (H_2_SO_4_) and hydrogen peroxide (H_2_O_2_) in a ratio of 3:1 to remove the passivation layer at 80 °C; (b) sputter aluminum nitride (AlN) to a thickness of 1 μm on the foil as a button insulating layer because AlN is a good electrical insulator, has high thermal conductivity and a high compression resistance; (c) deposit a 400 Å-thick chromium (Cr) layer onto the AlN layer as an adhesive layer using an E-beam evaporator, and evaporate a 2,000 Å gold (Au)-thick layer as a sensing layer; (d, e) use lithography and wet etching to form the sensing patterns of the multi-functional micro sensors; (f) use a spin coater to coat DURIMIDE^®^ 7505 polyimide; (g) use double-side lithography to form a mask layer to protect the stainless steel foil before etching the AlN and stainless steel foil; (h, i) use phosphoric acid and aqua regia to etch the AlN and stainless steel foil, respectively; (j) coat with a photoresist to define the top isolating layer to protect the micro sensors and fuel cells during gas shock in fuel cell testing.

**Figure 5.**
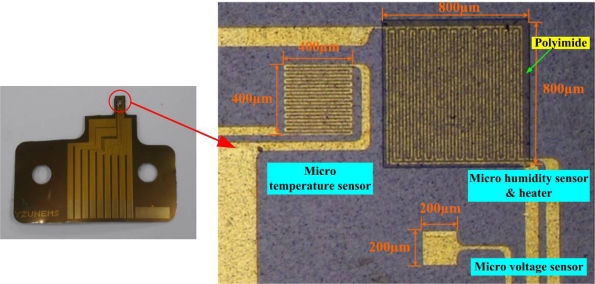
Optical microscopic photograph of multi-functional micro sensor.

Figure 5 shows the flexible multi-functional micro voltage, temperature and humidity sensors, with areas of 200 μm × 200 μm, 400 μm × 400 μm and 800 μm × 800 μm, respectively.

## 4. Experimental Results and Discussion

### 4.1. Calibration of Micro Temperature and Humidity Sensors

**Figure 6.**
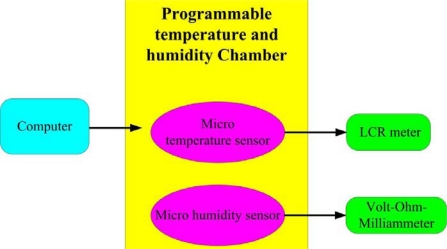
Temperature and humidity calibration system.

After the flexible multi-functional micro sensors were fabricated, they were connected via printed circuit boards using a wire bonder. The proposed micro sensors were calibrated in a Hungta HT-8045A programmable temperature and humidity chamber. Then, the signals from the multi-functional micro sensors were used to determine the variation of resistance and capacitance using an LCR meter, respectively. Figure 6 depicts schematically the temperature and humidity calibration system.

**Figure 7.**
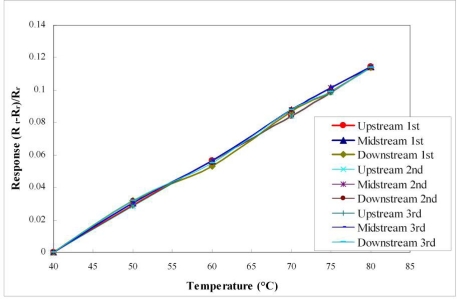
Calibration curves of micro temperature sensors upstream, midstream and downstream.

**Figure 8.**
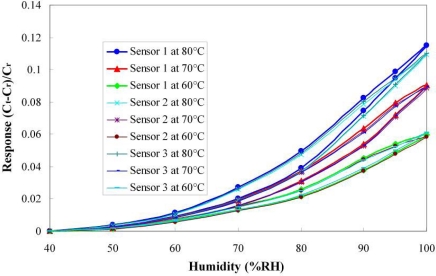
Calibration curves of micro humidity sensors at 60 °C, 70 °C and 80 °C.

**Figure 9.**
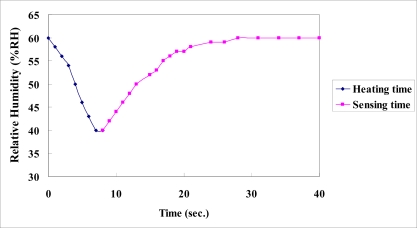
Response time of micro humidity sensor when used with a micro heater.

Figure 7 plots the calibration curves of the micro temperature sensors upstream, midstream and downstream. It reveals that the temperature sensors have high linearity and a sensitivity of 2.7 × 10^−3^ °C^−1^, based on three calibrations. Figure 8 plots the calibration curves of the micro humidity sensors at 60 °C, 70 °C and 80 °C. The calibrated results demonstrate that the proposed micro humidity sensors provided high accuracy and reproducibility, based on three calibrations. The micro humidity sensor alone requires a long response time, but the home-made micro humidity sensors have a shorter response time of 30 s, because they are used with a micro heater, as displayed in Figure 9.

### 4.2. Fuel Cell Testing

**Figure 10.**
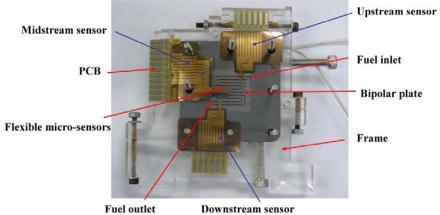
Proposed device embedded in PEM fuel cell.

**Figure 11.**
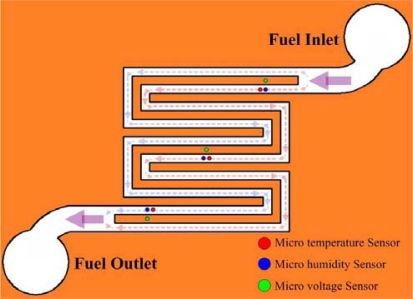
Locations of micro sensors.

The flexible multi-functional micro sensors were embedded in the cathode flow channel of the PEM fuel cell, as shown in Figure 10. Figure 11 displays the locations of the micro voltage, temperature and humidity sensors.

**Figure 12.**
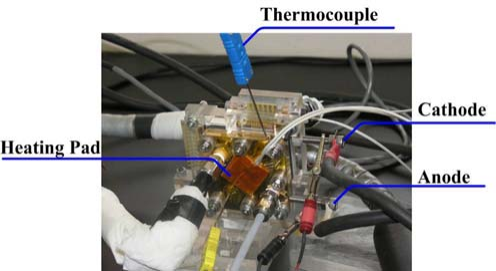
Fuel cell testing system.

In this work, variations in voltage, temperature, humidity and cell performance were measured using a fuel cell test station (850C) during the operation of a 100 W/A fuel cell, as shown in Figure 12. Table 1 lists the operating conditions and specifications of the fuel cell, including its temperature, relative humidity, current density, flow rate, flow-channel dimensions and reaction area. Experiments were conducted on the fuel cell in a steady state, and all tested parameters were returned to their initial values before each test, by purging with nitrogen.

#### 4.2.1. Comparison of Polarization Curves with and without Flexible Multi-Functional Micro Sensors

**Figure 13.**
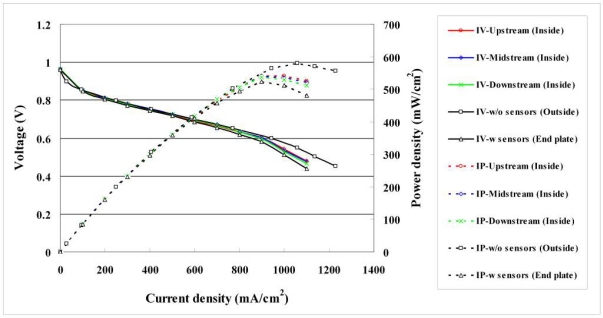
Comparison of polarization curves.

Figure 13 plots the cell performance curves without and with micro sensors upstream, midstream and downstream. The maximum power density without micro sensors is 579 mW/cm^2^, and that with micro sensors is 539 mW/cm^2^. The micro sensors thus degrade the performance by around 7%, since the masked area of micro sensors blocks the path of the reaction fuel. Table 2 compares the reaction area and power density without and with micro sensors. The performance upstream is better than that midstream, which is better than that downstream. The figure also reveals that the voltages measured at the end plate and inside the fuel cell differed more when the micro sensors were installed because the contact resistance was higher.

#### 4.2.2. Variation of Voltage, Temperature and Humidity at Different Relative Humidity and Constant Current Density of 0.1 A/cm^2^

**Figure 14.**
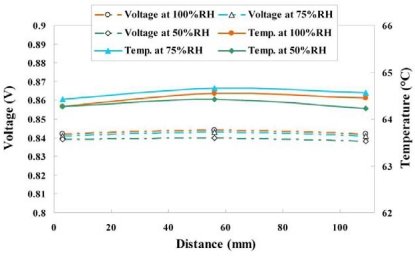
Variation of voltage and temperature at upstream, midstream and downstream (0.1 A/cm^2^).

**Figure 15.**
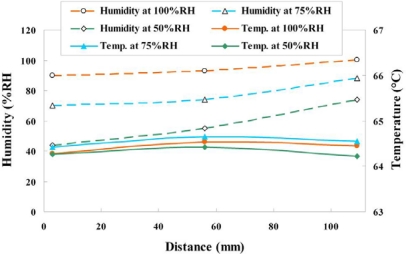
Variation of temperature and humidity at upstream, midstream and downstream (0.1 A/cm^2^).

Figures 14 and 15 plot variations of voltage, temperature and humidity at a constant current density of 0.1 A/cm^2^. According to these figures, the distributions of temperature and voltage are uniform upstream (3 mm), midstream (56 mm) and downstream (109 mm), because of a mitigating reaction, which does not clearly influence the voltage and temperature variations.

**Figure 16.**
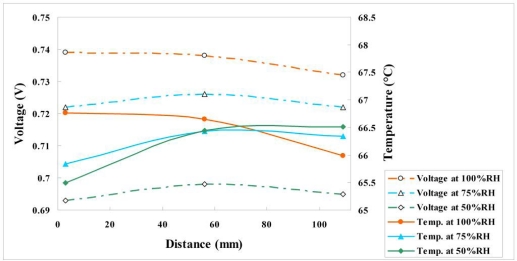
Variation of voltage and temperature at upstream, midstream and downstream (0.5 A/cm^2^).

Figure 16 reveals that the relative humidity increased slightly from the upstream, through the midstream to the downstream positions.

#### 4.2.3. Variation of Voltage, Temperature and Humidity at Different Relative Humidity and Constant Current Density of 0.5 A/cm^2^

**Figure 17.**
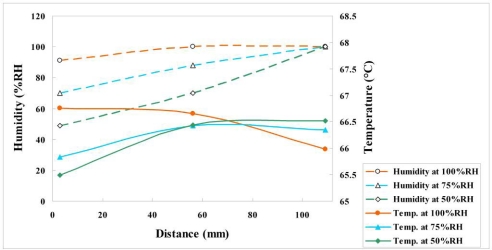
Variation of temperature and humidity at upstream, midstream and downstream (0.5 A/cm^2^).

Figures 16 and 17 plot the variations of voltage, temperature and humidity at a constant current density of 0.5 A/cm^2^. The experimental results at relative humidities of 75% and 50% indicate that the temperatures midstream and downstream exceed that upstream. Those at a relative humidity of 100% reveal that the local temperature downstream is less than that midstream and upstream, because flooding with water begins occurs downstream, affecting the reaction in the fuel cell. Water flooding is also responsible for the low voltage downstream.

#### 4.2.4. Variation of Voltage, Temperature and Humidity at Different Relative Humidity and Constant Current Density of 1 A/cm^2^

**Figure 18.**
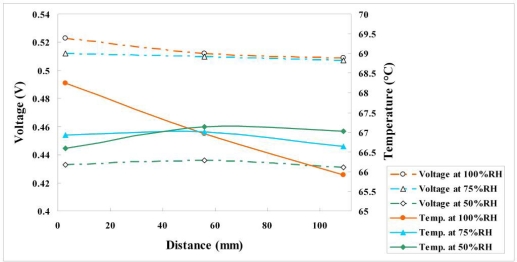
Variation of voltage and temperature at upstream, midstream and downstream (1 A/cm^2^).

**Figure 19.**
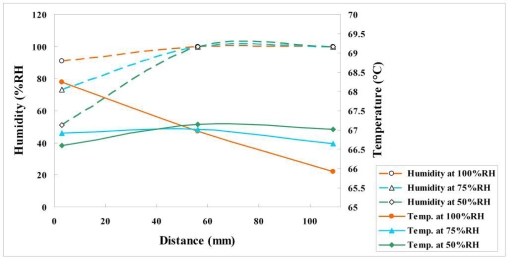
Variation of temperature and humidity at upstream, midstream and downstream (1 A/cm^2^).

Figures 18 and 19 plot the variations of voltage, temperature and humidity at a constant current density of 1 A/cm^2^. Figure 19 demonstrates that the voltages at relative humidities of 100% and 75% exceed that at a relative humidity of 50%, indicating that the proposed fuel cell performs well at relative humidities of 75% and 100%. At a relative humidity of 100%, the local temperature upstream is 1.3 °C higher than that midstream, because the fuel upstream is sufficient, and the local temperature midstream is 1 °C higher than that downstream, because of serious flooding downstream. The experimental results obtained at high current density and various values of relative humidity indicate that the relative humidity saturated both midstream and downstream, because the violent internal reaction caused serious water flooding there.

## 5. Conclusions

In this study, micro voltage, temperature and humidity sensors that are integrated on a stainless steel foil are fabricated using micro-electro-mechanical systems (MEMS). Home-made flexible multi-functional micro sensors have numerous advantages over conventional sensors, such as high temperature resistance, smallness, and high sensitivity. Most importantly, they can be placed anywhere in PEMFCs, and are suitable for use in the electrochemical environment of fuel cells.

Polyimide was coated on interdigitated electrodes as a humidity-sensing film and micro heaters with a serpentine structure was fabricated. Its design not only enhanced the sensitivity of micro humidity sensors but also reduced the response time. The calibrated results demonstrate that the proposed micro humidity sensors provided excellent accuracy and reproducibility.

A novel and feasible approach for *in-situ* measurement of local voltage, temperature and humidity in PEMFCs using flexible multi-functional micro sensors is established. The voltages measured at 100%RH and 75%RH exceed those measured at 50%RH, suggesting outstanding performance between 75%RH and 100%RH at a constant current density of 1 A/cm^2^. The experimental results obtained at a high current density and relative humidities of 100% and 75% demonstrate that the relative humidity saturated both midstream and downstream, because the violent internal reaction causes serious water flooding there.

## Figures and Tables

**Table 1.** Operating conditions and specifications of PEM fuel cell.

**Items**	**Conditions**
Cell temperature (°C)	65
Relative humidity (%RH)	50, 75, 100
Current density (A/cm^2^)	0.1, 0.5, 1
H_2_ flow rate (Anode) (sccm)	76 (λ = 2x at 1 A/cm^2^)
Air flow rate (Cathode) (sccm)	181 (λ = 2x at 1 A/cm^2^)
Bipolar plate/ Flow field type	Graphite/ Dual-path serpentine
Flow-channel depth (mm)	1.1
Flow-channel width (mm)	1.1
Flow-rib width (mm)	1.1
Reaction area (cm^2^)	5.29

**Table 2.** Comparison of reaction area, power density without and with micro sensors.

	**Reaction area**	**Power density at 0.7 V**	**Maximum power density**

**With micro-sensors**	4.76 cm^2^ (reduce 10%, 15 micro sensors)	452 mW/cm^2^ (degrade 5%)	539 mW/cm^2^ (degrade 7%)
**Without micro-sensors**	5.29 cm^2^	476 mW/cm^2^	579 mW/cm^2^
